# Trehalose synthesis in *Aspergillus niger*: characterization of six homologous genes, all with conserved orthologs in related species

**DOI:** 10.1186/1471-2180-14-90

**Published:** 2014-04-11

**Authors:** Åsa Svanström, Martin Richard van Leeuwen, Jan Dijksterhuis, Petter Melin

**Affiliations:** 1 Uppsala BioCenter, Department of Microbiology, Swedish University of Agricultural Sciences, P.O. Box 7025, SE-750 07 Uppsala, Sweden; 2 Applied and Industrial Mycology, CBS-KNAW Fungal Biodiversity Centre, Uppsalalaan 8, 3584 CT Utrecht, The Netherlands; 3 Present address: Swedish Chemicals Agency, Box 2, SE-172 13 Sundbyberg, Sweden

**Keywords:** Ascomycota, Conidia, Germination, *Saccharomyces cerevisiae*, Stress-resistance, Targeted gene deletion

## Abstract

**Background:**

The disaccharide trehalose is a major component of fungal spores and is released upon germination. Moreover, the sugar is well known for is protective functions, e.g. against thermal stress and dehydration. The properties and synthesis of trehalose have been well investigated in the bakers’ yeast *Saccharomyces cerevisiae*. In filamentous fungi, such knowledge is limited, although several gene products have been identified.

**Results:**

Using *Aspergillus niger* as a model fungus, the aim of this study was to provide an overview of all genes involved in trehalose synthesis. This fungus has three potential trehalose-6-phosphate synthase encoding genes, *tpsA*-*C*, and three putative trehalose phosphate phosphatase encoding genes, *tppA*-*C*, of which two have not previously been identified. Expression of all six genes was confirmed using real-time PCR, and conserved orthologs could be identified in related Aspergilli. Using a two-hybrid approach, there is a strong indication that four of the proteins physically interact, as has previously been shown in *S. cerevisiae*. When creating null mutants of all the six genes, three of them, *ΔtpsA*, *ΔtppA* and *ΔtppB*, had lower internal trehalose contents. The only mutant with a pronounced morphological difference was *ΔtppA*, in which sporulation was severely reduced with abnormal conidiophores. This was also the only mutant with accumulated levels of trehalose-6-phosphate, indicating that the encoded protein is the main phosphatase under normal conditions. Besides *ΔtppA*, the most studied deletion mutant in this work was *ΔtppB*. This gene encodes a protein conserved in filamentous Ascomycota. The *ΔtppB* mutant displayed a low, but not depleted, internal trehalose content, and conidia were more susceptible to thermal stress.

**Conclusion:**

*A. niger* contains at least 6 genes putatively involved in trehalose synthesis. Gene expressions related to germination have been quantified and deletion mutants characterized: Mutants lacking *tpsA*, *tppA* or *tppB* have reduced internal trehalose contents. Furthermore, *tppA,* under normal conditions, encodes the functional trehalose-6-phosphate-phosphatase.

## Background

Trehalose (α-D-glucopyranosyl-α-D-glucopyranoside) is a non-reducing disaccharide that is present in a wide variety of organisms. It has been isolated from plants, fungi, nematodes and insects [1-3]. In fungi, trehalose has been shown to accumulate in dispersal and survival structures such as spores (where it can constitute as much as 10% of the dry weight), sclerotia, and in yeast cells going into stationary phase [3,4] . Since the sugar is rapidly degraded when these structures germinate or resume vegetative growth, early research concluded that trehalose serves as a storage molecule [5,6]. However, later studies showed that the function of trehalose is more complex and diverse than just serving as an energy reserve; the molecule has been shown to function as a regulator of carbon metabolism [1], a signaling molecule and a protection molecule against various kinds of abiotic stress [3,7]. Several fungal species have been shown to induce trehalose production as a stress response. Examples include: *Saccharomyces cerevisiae*[8,9], *Zygosaccharomyces bailii*[10], *A. nidulans*[11], *A. fumigatus*[12], *Rhizopus oryzae*[13], and *Botrytis cinerea*[14]. Trehalose is known to protect both proteins and lipid membranes of living cells against stressors such as heat, desiccation and cold. Although the mode of bio-protection of trehalose is not fully elucidated, three main hypotheses are generally accepted, and the true mechanism is likely a combination of these. The hypotheses include: water replacement (direct interaction of trehalose with the protected structure through hydrogen bonds); mechanical entrapment (glass formation of trehalose that creates a protective coating around the structure); preferential exclusion (bulk water is ordered around trehalose and is thereby separated from the bio-molecule, which then becomes more compact and stabilized) [15,16]. The physico-chemical properties of trehalose that lie behind these hypotheses include several crystalline forms, a high glass transition temperature, and the stereochemistry of the sugar [7,15].

In fungi, trehalose is synthesized via the intermediate trehalose-6-phosphate (T6P) and involves two enzymatic steps. First, T6P is formed from one glucose-6-phosphate and one UDP-glucose catalyzed by T6P-synthase (here called TPS). In the next step, the phosphate molecule is removed by trehalose-phosphate-phosphatase (here called TPP) yielding trehalose [1,11]. The organism in which trehalose synthesis has been most thoroughly studied is *S. cerevisiae*. Here, four homologous gene products responsible for trehalose synthesis physically interact forming a “trehalose synthase complex”, which consists of one TPS (called Tps1), one TPP (called Tps2), and two other subunits, Tsl1 and Tps3*,* with proposed regulatory and stabilizing functions [6,17-19]. In filamentous fungi, the gene products involved in trehalose synthesis are not as thoroughly investigated as in *S. cerevisiae*, but have been studied with respect to germination [20], plant pathology [21] and human pathology [12,22].

Within Aspergilli, several individual gene products have been identified and characterized. In *A. niger*, two Tps1 orthologs, *tpsA* and *tpsB*, have been identified and characterized. At ambient temperature, the trehalose level of *ΔtpsA* mycelia was lowered compared to wild-type. In contrast to the constitutively expressed *tpsA*, the expression of *tpsB* was induced by thermal stress [23]. In the opportunistic human pathogen *A. fumigatus*, four Tps1 paralogs, *tpsA* – *D* have been identified [12]*.* When deleting these genes, the authors found that either *tpsA* or *tpsB* was sufficient to maintain normal trehalose levels, but if both genes were deleted, the resulting mutant strain was depleted of trehalose and showed slower germination rates as well as higher susceptibility to heat and oxidative stress compared to wild-type. Another notable finding was that this double mutant was hypervirulent in infected mice [12]. In *A. nidulans*, a Tps1 ortholog, *tpsA*, has been identified and deleted. In this mutant, trehalose was not accumulated, and in addition, the authors could conclude that in *A. nidulans* trehalose is important for resistance to continual exposure to sub-lethal stress but not to short exposure of lethal stress [11]. In contrast to *S. cerevisiae*, *tps* mutants in Aspergilli are able to utilize glucose as carbon source [11,23,24]. All identified Tps1 orthologs in Aspergilli are generally much shorter than the *S. cerevisiae* Tps1, around 500 amino acids compared to 1447.

Besides Tps1 orthologs, two Tps2 orthologs have been identified within the Aspergilli, one in *A. nidulans*[25] and one in *A. fumigatus*[22]: In both species they are designated *orlA*. The *ΔorlA* mutant of *A. fumigatus* had a pronounced phenotype with abolished asexual reproduction as well as decreased virulence. However, the phenotype could be restored to wild-type appearance by growing the mutant on media containing an osmotic stabilizer (sorbitol or glycerol). As also observed in *A. nidulans*, the *A. fumigatus ΔorlA* mutant strain contained wild-type levels of trehalose but the T6P levels were elevated [22,25].

In this study we focused on trehalose synthesis in filamentous fungi, and more specifically, in *Aspergillus niger*. This is a common food spoilage mould as well as an industrially important organism, utilized for production of citric acid, for instance [26]. Six genes, *tpsA* (ANI_1_1406074)*, tpsB* (ANI_1_1078064)*, tpsC* (ANI_1_1216124)*, tppA* (ANI_1_1432094)*, tppB* (ANI_1_48114) and *tppC* (ANI_1_2070064) were identified to be involved in trehalose biosynthesis. Expression of these genes was studied during conidial outgrowth. In addition, we deleted these genes and characterized the mutants in terms of trehalose and T6P content, protein interactions, and stress survival coupled to situations often occurring in foodstuff.

## Methods

### Software, hardware and computer-based analyses used in this study

GraphPad Prism® version 5 was used for generating figures (line drawings) and calculating mean, standard error of the mean, and significance between samples (using one or two way ANOVA and Bonferroni post-test). Adobe Illustrator CS5 and Adobe Photoshop CS6 were used for managing pictures (cropping and minor changes in contrast levels for best visualization). Bio-Rad CFX 96™ Real-Time System was used for generating gene expression data and the Bio-Rad CFX Manager™ version 1.6 software was used for analyzing the data. MacVector version 12 was used for primer design and phylogenetic analyses.

### Culture maintenance, spore preparation and spore densities

The fungal strains used in this study are listed in Table 1. As wild-type, we used the *A. niger* N402 strain, which is also the mother strain of all generated mutants [27]. The strains were maintained on *Aspergillus* Minimal Media (AMM) as previously described [28]. For the MA70.15 and MA169.4 strains, AMM was supplemented with 10 mM uridine. The complemented strain (*tppB+*) was maintained on AMM containing Hygromycin B (0.10 mg/ml). The *ΔtppA* mutant was tested for sporulation both on AMM agar and on AMM agar containing 1.2 M sorbitol. Normally plates were incubated at 25°C for 14 days. All deletion mutants as well as the control strains were tested for growth in 10, 15, 20, 25, 30 and 37°C for 14 days. For trehalose measurements, conidia were harvested from plates incubated at 25°C for 5, 14, 28 and 90 days. Spore suspensions were prepared in water containing Tween 80 (0.01% v/v), were filtered through sterile Miracloth (Calbiochem), and the spore count was determined using a Bürker chamber. To estimate the number of conidia produced, a circular area of 95 mm^2^ was cut out from centrally inoculated AMM plates that had been incubated at 25°C for 14 days. 10 ml of water containing Tween 80 (0.01% v/v) and 10 glass beads (2 mm in diameter) were added to the agar plug, the mixture was vortexed for 10 min and spore concentrations were counted in a Bürker chamber. Three biological replicates, each calculated from the average of three technical replicates, were used for all samples.

**Table 1:** Strains used in this study

**Strain**	**Genotype**	**Reference**
N402	*cspA1*	[27]
MA70.15	*cspA1,pyrG1,* ∆*kusA::amdS*^*+*^	[32]
MA169.4	*cspA1,pyrG1,* ∆*kusA::DR-amdS*^*+*^*-DR*	[33]
J699* (∆*tpsA*)	*cspA1,pyrG1,* ∆*kusA::amdS*^*+*^, ∆*tpsA::pyrG*	This study
J700 (∆*tpsB*)	*cspA1,pyrG1,* ∆*kusA::amdS*^*+*^, ∆*tpsB::pyrG*	This study
J701 (∆*tpsC*)	*cspA1,pyrG1,* ∆*tpsC::pyrG*	This study
J684 (∆*tppA*)	*cspA1,pyrG1,* ∆*kusA::amdS*^*+*^, ∆*tppB::pyrG*	This study
J685 (∆*tppB*)	*cspA1,pyrG1,* ∆*kusA::amdS*^*+*^, ∆*tppB::pyrG*	This study
J702 (∆*tppB2*)	*cspA1,pyrG1,* ∆*tppB::pyrG*	This study
J686 (∆*tppC*)	*cspA1,pyrG1,* ∆*kusA::amdS*^*+*^, ∆*tppC::pyrG*	This study
J689 (*pyrG*+)	*cspA1,* ∆*kusA::amdS*^*+*^	[28]
J693 (t*ppB*+)	*cspA1,pyrG1,* ∆*tppB::pyrG, tppB::hph*	This study

*Strain numbers from the fungal collection at the Department of Microbiology, Swedish University of Agricultural Sciences.

### Low-temperature scanning electron microscopy (SEM)

Wild-type, N402, and *ΔtppA* were grown for 1 week on AMM. Margins of colonies containing conidiophores were excised with a surgical blade and carefully transferred into a copper cup (diameter 10 mm, height 8 mm). Dislodging during snap freezing was prevented by gluing agar blocks in the copper cup with frozen tissue medium (KP-Cryoblock, Klinipath, Duiven, the Netherlands). The sample was snap-frozen in nitrogen slurry and immediately transferred in a vacuum transfer device to an Oxford CT1500 Cryostation attached to a JEOL 5600LV scanning electron microscope (JEOL, Tokyo, Japan). Electron micrographs were acquired from uncoated frozen samples, or after sputter-coating with gold three times during 30 s. Micrographs of uncoated samples were taken at an acceleration voltage of 2.5 kV, and consisted of 30 averaged fast scans (SCAN 2 mode). Coated samples were observed at 5 kV using F4 scans.

### Extraction of nucleic acids

DNA was extracted as previously described [28]. RNA from dormant conidia and conidia in early stages of germination (0 and 3 h) was extracted according to Leeuwen and co-workers [29]. RNA from germinating spores (6 and 12 h), mycelia and sporulating mycelia (plate) were extracted according to Plumridge and co-workers [30]. As a final step in both protocols, the RNA products were purified using a Qiagen RNeasy Mini kit (RNA clean up protocol).

### Two-hybrid assay

The two-hybrid assay was performed using the BACTH System Kit (Euromedex). Full-length cDNA for all six genes were amplified using primers with internal restriction sites (Table 2). After digestion of the PCR products, the inserts were ligated into linearized and dephosphorylated pKT25 and pUT18C vectors and used to transform *E. coli*. All ligations in this work were performed with the ReadyToGo ligation kit (GE Healthcare) and were transformed into NEB 10-β Competent *E. coli* cells (New England Biolabs), unless otherwise stated. Correct insertions were confirmed with vector specific primers (Table 2) followed by sequencing. Successful clones were co-transformed into electrocompetent BTH101 cells and selected on LA plates supplemented with ampicillin (100 μg/ml) and kanamycin (50 μg/ml). The protein-protein interactions were assayed according to the manufacturer’s protocol with the following modifications. One fresh colony of each interaction was transferred to 100 ml conical flasks with 5 ml LB supplemented with ampicillin 50 μg/ml, kanamycin 50 μg/ml and 0.5 mM IPTG, and incubated with shaking at 100 rpm at 20°C for 72 h. The extent of protein-protein interaction was measured with β-galactosidase assays as units/mg dry weight.

**Table 2:** Primers used for cDNA synthesis, qPCR and Two-Hybrid cloning

**Primer name**	**Sequence 5′-3′**	**Purpose**
T12VN	TTTTTTTTTTTTVN	cDNA synthesis
tpsAF	TGAGGGCTGTTGTGAATGAGC	qPCR *tpsA*
tpsAR	ACTCGGAAAGCACCAAGACACC	
tpsBF	GTGGGCAGAATCAACGGAAAG	qPCR *tpsB*
tpsBR	TGAACACTTGGATAGTTCGGCAAC	
tpsCF	TTGCCGATGCCTGCTTGTTG	qPCR *tpsC*
tpsCR	TTCGCTGGATGGAAAGTAAGACAC	
tppAF	TTGAAGACACCGTTGGGAAGAG	qPCR *tppA*
tppAR	GGAGCAAAAGATGAACTCAGGAGC	
tppBF	TGGACACTTACCTCTGGGATGAAG	qPCR *tppB*
tppBR	GCTGATGGGCATTGAGTATTTCC	
tppCF	AAAGCCAAAGCAGCCGAATC	qPCR *tppC*
tppCR	TGCCCGTTAGTATCCTCAGCAGAG	
actF	TCGTGACCTGACGGATTACCTC	qPCR actin
actR	TGGAAGAAGGAGCAAGAGCAGTG	
pKT25F	ACGATTTCGAGGCGGTCAAG	Confirmation of cloned cDNA to pKT25 vector
pKT25R	GATGTGCTGCAAGGCGATTAAG	
pUT18CF	TGTCTTCTACGAGAACCGTGCATAC	Confirmation of cloned cDNA to pUT18C vector
pUT18CR	CGGTGAAAACCTCTGACACATGC	
tpsAFpst	GACTCTGCAGCCGTTTCCGACAGCATGCCTT	Cloning of *tpsA* cDNA
tpsAFbam	TATCTGGATCCCGTTTCCGACAGCATGCCTT	
tpsARkpn	TGATCGGTACCAGCTCACTGTGCCACCTGCT	
tpsBFbam	ATCAGGATCCTCCTTTTCCAATGGCTGCCAA	Cloning of *tpsB* cDNA
tpsBeco	ATCAGAATTCAGCTGCAGTCATAACATAATCA	
tpsCFxba	AGTCTCTAGAATCCAGAATGACGAAGCGCAA	Cloning of *tpsC* cDNA
tpsCReco	ACTCGAATTCCGTCCATGTCAGGGCTCAA	
tppAxbaF	ACTGTCTAGAATCCCCCATCATGCT	Cloning of *tppA* cDNA
tppAecoR	AGTAGAATTCATTAACAGAACCCTCAATAC	
tppBxbaF	TACATCTAGATGTCGCCATGACCATCTACA	Cloning of *tppB* cDNA
tppBkpnR	TTCCGGTACCCCTTTCACTCCTTATCGTGA	
tppCFsal	TATCGTCGACCACCCCAATGACGGTCTTCAT	Cloning of *tppC* cDNA
tppCRbam	CATAGGATCCTCAGTCATGGCTTTCTCCGT	

### cDNA synthesis and Real-Time PCR

Using total RNA as template, cDNA was synthesized using a polyT primer (Table 2) and the enzyme SuperscriptIII (Invitrogen) according to the manufacturer’s protocol. Quantitative real-time PCR was performed with the BioRad CFX-96 system using the EvaGreen reagent (BioRad), gene specific primers (Table 2), and the following protocol: Initial denaturation and enzyme activation, 95°C 30 s; 40 cycles of 95°C for 2 s and 56-60°C for 8 s; plate read; and finally, melt curve analysis starting at 65°C and ending at 95°C. Relative expression for *tpsA-C* and *tppA-C* were calculated from and compared to a serially-diluted cDNA pool and normalized to the actin-encoding gene (ANI_1_106134), which has been successfully used in previous experiments [28,31] and is expressed at high levels throughout germination according to published microarray data [29]. For each growth stage, the expressions were calculated from four biological replicates, each with three technical replicates. To verify the expression, or lack thereof, in the reconstituted and null mutant of *tppB*, the expression in mutants was normalized against N402 as previously described [28] using the efficiency calibrated mathematical method for the relative expression ratio in real-time PCR [32].

### Gene deletions and complementation

Deletion constructs for the genes, *tpsA, tpsB, tppA, tppB and tppC* were made using fusion PCR to replace the coding sequence with the *A. oryzae pyrG* gene, and used to transform the uridine auxotrophic strain MA70.15 [33] as previously described [29]. With the same technique, a mutant lacking both *tpsB* and *tppC* was created. A second deletion mutant of *tppB*, (*ΔtppB2*) was generated in a different uridine auxotrophic strain, MA169.4 [34]. Both MA70.15 and MA169.4 have deficient *kusA* that is the *A. niger* ortholog of kus70, which is required for the non-homologous end-joining pathway [35]. The *tpsC* deletion strain was constructed by cloning *tpsC* in the standard pBS-SK vector (Stratagene) using *Bam*HI and *Xho*I. Next, the vector was digested with *Hin*dIII to remove 1648 bp, containing most of the coding sequence. After dephosphorylation of the vector, a *Hin*dIII digested PCR product of the *A. oryzae pyrG* gene was ligated into the vector, thus replacing *tpsC*. This deletion construct was PCR-amplified and used to transform strain MA169.4. All *A. niger* transformants were confirmed using PCR and sequencing. For the deletion strains where MA169.4 was used as parent strain, the *kusA* gene was repaired using induced recombination by repeated transfer to agar plates supplemented with fluoroacetamide 0.75 μg/ml, as described [34]. All primers for gene deletions are listed in Table 3. The *ΔtppB* strain was complemented as previously described [28]. Briefly, the strain was transformed with a plasmid carrying an intact copy of *tppB* and a cassette carrying hygromycin resistance.

**Table 3:** Primers used for targeted gene deletions

**Primer name**	**Sequence 5′-3′**	**Purpose**
pyrGN2	CACATGCCTCATTTTGACCA	Mutant confirmation
PyrtpsAup	ACCGTTGGAAGGTGGGATCCTATGGATCTCAGAA	Amplifies *pyrG* with 3' *tpsA* overhangs
PyrtpsAdown	CCTTTCAGAATGAGTGTGAGCGGATAACAATTTC	
tpsAup	CCATCTGTCTAGCTCTTCATCCCC	*tpsA*, upstream fragment
tpsApyrup	GATCCATAGGATCCCACCTTCCAACGGTGTAGAGACTCC	
tpsApyrdown	TTATCCGCTCACACTCATTCTGAAAGGTGGGGTTTTC	*tpsA*, downstream fragment
tpsAdown	GCAAGATTCCCGCATCCATC	
tpsAupN1	CAACCCCACCAGTTCTCTCAAG	Amplification of KO-fragment
tpsAdownN1	AAAGGGAGTTCCAAGCAGCCTG	
pyrtpsBup*	ATCTGCTCTGCCTGGGATCCTATGGATCTCAGAA	Amplifies *pyrG* with 3′ *tpsB* overhangs
pyrtpsBdown	CTGCCCATCACCATGTGAGCGGATAACAATTTC	
tpsBup*	TTGAACCCTTGAAACCGAACAC	*tpsB*, upstream fragment
tpsBpyrGup*	GATCCATAGGATCCCAGGCAGAGCAGATACTTACCCGTC	
tpsBpyrGdown	TTATCCGCTCACATGGTGATGGGCAGACGATTG	*tpsB*, downstream fragment
tpsBdown	TGCTAAAGAGGGTGTGGGATTG	
tpsBupN3	TCCCGATTGGTAGAATCCCTAAAG	Amplification of *tpsB* KO-fragment
tpsBdownN3	CATGCGAAAATGACAGGAACATTC	
pyrGuphind	TAAAAGCTTCTATATTGATCCTTA	*pyrG*, KO of *tpsC*
pyrGdown	TGTGAGCGGATAACAATTTC	
tpsCupN-2	TGCCGAATTGACGTGCGTAGAG	Cloning of *tpsC*
tpsCdownN-2	TGGTGGTGAACCTTTCGTTGTTC	
tpsCupN5	CCCTCCATACTTACTCCATACATCTCG	Amplification of *tpsC* KO-fragment
tpsCdownN5	CCAGCTTGACACATCCAACATAAC	
pyrtppAup	CCTGTCCCCGCTTCAAGAAAGGGATCCTATGGATCTCAGAA	*pyrG* with 3′ *tppA* overhangs
pyrtppAdown	GAGTCATCAGTGCTGCTTTCTGCTGTGAGCGGATAACAATTTC	
TppAup	TGTTGGAAGCGTCTTTCTGCC	*tppA*, upstream fragment
tppApyrup	TTCTGAGATCCATAGGATCCCTTTCTTGAAGCGGGGACAGG	
tppApyrdown	GAAATTGTTATCCGCTCACAGCAGAAAGCAGCACTGATGACTC	*tppA*, downstream fragment
tppAdown	TGTCCGATTGGGGGTGATTG	
tppAupN1	TGAGGAGGCGTTGTCAAAAGATAG	Amplification of *tppA* KO-fragment
tppAdownN1	CGATTGGGGGTGATTGGCTTAC	
pyrtppBup	CGGTAGGTTAGGGATCCTATGGATCTCAGAA	Amplification of *A. oryzae pyrG* with 3′ *tppB* overhangs
pyrtppBdown	GTTTGTCTTGTGTGAGCGGATAACAATTTC	
tppBup	ATACCAAGCAATCGCCCAAGCCAG	*tppB*, upstream fragment
tppBpyrGup	TCCATAGGATCCCTAACCTACCGCCCAAAGAGAGAGC	
tppBpyrGdown	TTGTTATCCGCTCACACAAGACAAACGATGCGGAATG	*tppB*, downstream fragment
tppBdown	CGTATCCTGGACTTTCAGCACG	
tppBupN1	TTTTCGACCTTGGTGGGTGCTTCC	Amplification of *tppB* KO-fragment
tppBdownN1	GAGACATTGTCGGTCAGTGAGGTAG	
pyrtppCup	TGTCCTTCAGGGATCCTATGGATCTCAGAA	*pyrG* with 3′ *tppC* overhangs
pyrtppCdown*	CTGTTCAGCATTGTGAGCGGATAACAATTTC	
tppCup	ATGAGGTGATAGTCGTGGACCCAG	*tppC*, upstream fragment
tppCpyrup	TCCATAGGATCCCTGAAGGACAAAGACAGGCTGAAG	
tppCpyrdown*	TTGTTATCCGCTCACAATGCTGAACAGATGATCCCCAG	*tppC*, downstream fragment
tppCdown*	TCGAGGTAGAGGTTCCCTTTCG	
tppCupN1*	CGATAGTCTTTGCGAACAGACGG	Amplification of *tppC* KO-fragment
tppCdownN1*	CGAGGTAGAGGTTCCCTTTCGATG	
tpsBupN1	CCCTTTCCCGATTGGTAGAATC	Amplification of *tpsB*/*tppC* double mutant

*Also used in the design of the *tpsB*/*tppC* double mutant.

### Extraction and quantification of trehalose and trehalose-6-phosphate

Trehalose from dormant and swollen conidia, germlings and mycelia was extracted and quantified as previously described [28]. In brief, harvested fungal material was freeze-dried and homogenized using a mortar. Samples were diluted with ultra pure water, boiled, evaporated and derivatized by trimethylsilylanization before injection into the gas chromatograph–mass spectrometer (GC–MS). Relative concentrations of α-α-trehalose were calculated as the ratio to an internal standard (α-β-trehalose) and thereafter correlated to a standard curve to obtain the absolute concentrations. All trehalose measurements were performed in biological duplicates based on the average of three technical triplicates.

Extraction and quantification of T6P was performed essentially as described by [22]. Liquid cultures were inoculated with 10^6^ spores per ml, incubated at 25°C for 3 days at 140 rpm, and all mycelia from one culture made up one sample. Three biological replicates based on the average of three technical replicates were used for all strains.

### Stress tolerance and long term viability of conidia

Dormant conidia from wild-type *A. niger*, the additional control strain *pyrG+*, and the deletion mutants *ΔtppB* and *ΔtppB*2 were subjected to heat stress for 20, 60, 90 and 120 min at 55°C. Dormant conidia of wild-type, *pyrG +* and *ΔtppB* were subjected to sub-lethal salt and benzoic acid stress by being spread on AMM plates containing benzoic acid or NaCl at concentrations ranging from non-effective to total growth inhibition of the control strains. For detailed description of these stress experiments see [28]. In addition, dormant conidia from control strains and *ΔtppB* were subjected to oxidative stress by adding 200 mM H_2_O_2_ to freshly made conidial suspensions (approximately 250 spores/ml liquid AMM). The suspensions were incubated for 10, 20 or 40 min before being spread on AMM plates. To test long-term viability, conidial suspensions (10^6^ conidia/ml water) were stored at 4°C for a total of 8 weeks. An aliquot of the suspension was withdrawn weekly, diluted and spread on AMM plates for enumeration.

Plates from all experiments were incubated at 25°C for 3–7 days before CFU were estimated, and all experiments were performed at least in triplicates (based on three technical replicates).

## Results

### Identification of genes involved in trehalose synthesis in *Aspergillus niger* and other fungi

**Figure 1:**
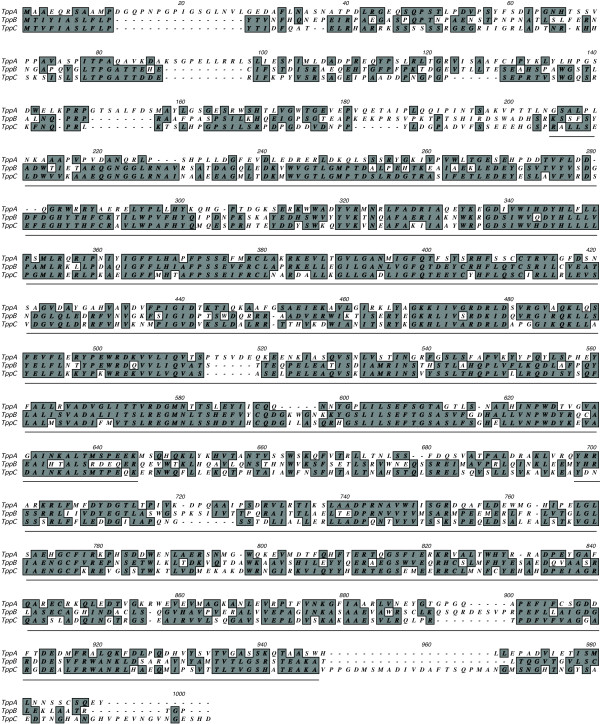
**Protein alignment indicating the similarities between the *A. niger *Tpp proteins.** Boxed amino acids are either identical or similar in at least two of the aligned sequences. Approximated borders of the phosphate synthase (closer to the N-terminal) and the phosphatase (closer to the C-terminal) domains are indicated in the figure.

Known amino acid sequences of the proteins of the trehalose synthesis complex of *S. cerevisiae* were used as queries to identify homologous genes in the *A. niger* genome by searching the databases available at NCBI using blastP (http://blast.ncbi.nlm.nih.gov). We confirmed the presence of two previously characterized tps1 orthologs: *tpsA* (ANI_1_1406074) and *tpsB* (ANI_1_1078064). Also, we could detect two genes, previously identified by sequence homology [36]; a third tps1 paralog, *tpsC* (ANI_1_1216124), and a tps2 ortholog, which we call *tppA* (ANI_1_1432094). In addition, we could identify two previously unidentified, putative *tppA* paralogs designated *tppB* (ANI_1_48114) and *tppC* (ANI_1_2070064). Compared to TppA, these two encoded proteins were of similar length (all three proteins have between 926 to 946 residues) and had a protein identity of 37% (250 out of 683) and 35% (241 out of 688), respectively (Figure 1). From the NCBI’s Conserved Domain Database [37] it was revealed that all three Tpp proteins contain a phosphate synthase domain approximately 200 residues from the N-terminal, and a phosphatase domain approximately 700 residues from the N-terminal (Figure 1). The Tps proteins only contain the phosphate synthase domain (data not shown). In summary, three tps1 orthologs, tpsA-C, and three tps2 orthologs, tppA-C, were identified from the *A. niger* genome.

**Figure 2:**
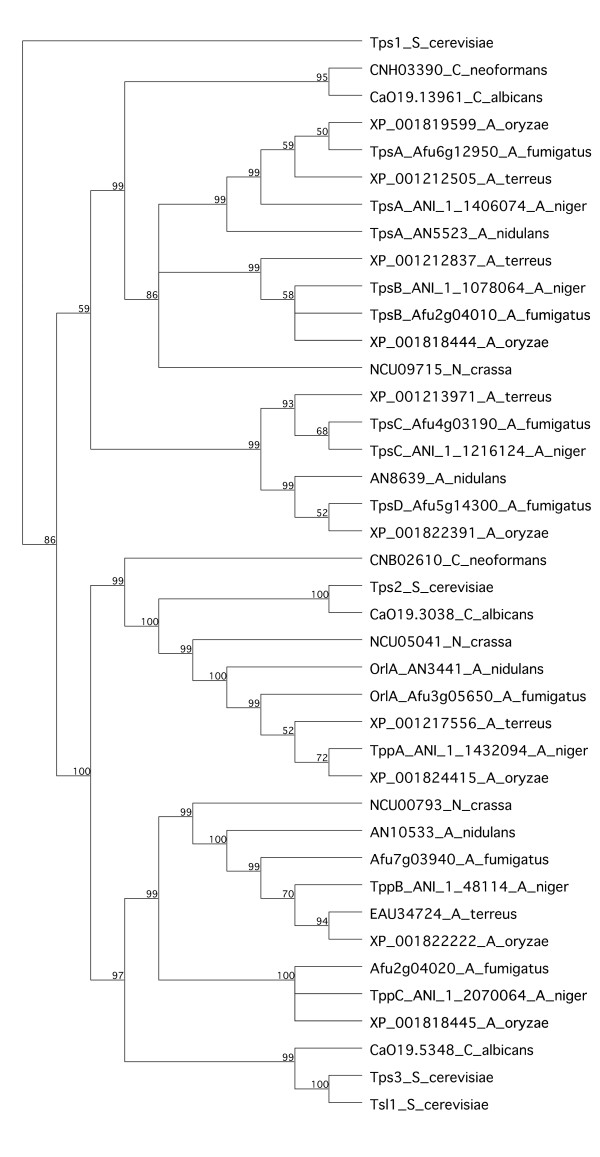
**Proteins in the trehalose synthesis family.** Analyzed species are: *A. fumigatus, A. nidulans, A. niger, A. oryzae, A. terreus, Candida albicans, Cryptococcus neoformans, Neurospora crassa* and *S. cerevisiae*. All proteins from filamentous fungi have their accession number included. For *A. niger* and previously characterized gene products, given names are also included. This phylogenetic tree was built using the neighbor joining algorithm with 32 000 bootstrap replicates. Based on sequence identities, the *S. cerevisiae* Tps1 protein was selected by the software as outgroup. Optional settings or use of other algorithms gave identical, or very similar, results.

The obtained amino acid sequences of Tpp and Tps proteins were compared to those present in all known genomes of *Aspergillus* species, as well as other fungal species as references. For this, we used blastP at NCBI (http://blast.ncbi.nlm.nih.gov) and AspND (http://www.aspergillusgenome.org/; [38]; available August 2013). All identified fungal genomes contain at least one putative T6P synthase and trehalose-6-phosphate-phosphatases orthologous to Tps2/TppA/OrlA. In addition, TppB could be identified in all filamentous Ascomycota, whereas TppC is only present in the *Aspergillus* subgenera Fumigati and Circumdati. Both TppB and TppC group together with the *S. cerevisiae* Tps3 and Tsl1 proteins. The relationships of different gene products in some reference species are displayed as a phylogenetic tree (Figure 2). An additional observation is that, whenever present, *tpsB* and *tppC* are located adjacent on the chromosomes. The protein outside the putative trehalose synthase complex that had the highest blast score against TpsA was ANI_1_512164, encoding a glutamate carboxypeptidase, where the most similar region consisted of 30% over 50 amino acid residues. In contrast, close homologs could be identified in more distantly related species such as the bacterium *Escherichia coli* and the protist *Dictyostelium discoideum* (data not shown).

### Two-hybrid assay to reveal putative protein-protein interactions

In order to determine whether the homologous proteins physically interact, as has been reported in *S. cerevisiae*[39], we performed a bacterial-based two-hybrid assay screening for interactions between all six *A. niger* proteins. For each protein, the full-length open reading frame was cloned into an expression vector and co-transformed into *E. coli* cells. All 36 possible combinations of *A. niger* proteins were screened, together with two clones containing different subunits of the leucine zipper GCN4 serving as a positive control and four combinations of one *A. niger* protein and one bacterial protein serving as negative controls. Results with no interactions were repeated at least once in an additional independent two-hybrid assay. Where interactions were detected, the assay was repeated in at least two independent assays. Results indicated that TpsB interacts with TpsA, TpsB and TppA, and that all Tps units interact with themselves (Table 4). All putative interactions involving either TppB or TppC did not score any signals above the negative controls (data not shown).

**Table 4:** Protein-protein interactions assayed by Bacterial adenylate cyclase two-hybrid system

**Protein**	**TpsA**	**TpsB**	**TpsC**	**TppA**
TpsA	**418** (210–863)*	**1746** (1582–1799)	113 (77–135)	71 (43–89)
TpsB	**1593** (1467–1832)	**1776** (1658–1988)	**441** (341–560)	**581** (322–714)
TpsC	172 (101–244)	**688** (315–980)	**1214** (861–1551)	80 (67–102)
TppA	429 (167–656)	**691** (462–987)	156 (133–198)	83 (58–98)

*Estimated values are in units/mg dry weight bacteria. Values in parentheses are the highest and lowest scores for each based on three to four independent assays. The positive control zip-zip (T18 and T25 fragments of the leucine zipper of GCN4) was scored to 3429 (2938–4270). Negative controls and remaining protein interactions scored at maximum 220 (zip-tpsA) but usually less than 50. Values in bold are considered true protein-protein interactions.

### Gene expression during conidial outgrowth

**Figure 3:**
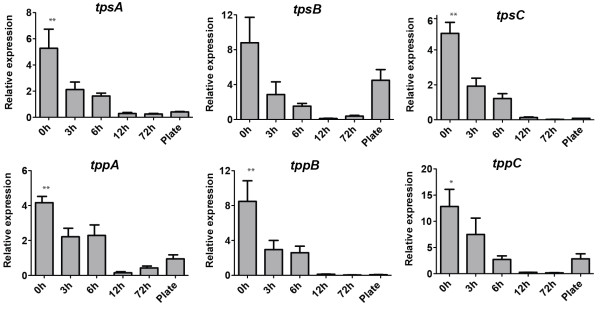
**Expression of putative trehalose synthesis genes during outgrowth of *A. niger *conidia.** The developmental stages are given on the *x*- axis: 0 h are dormant conidia; 3–72 h are swollen conidia, germlings or mycelia after so many hours of incubation in liquid AMM media; and Plate is the entire sporulating culture grown on AMM plates for 5 days. Error bars show standard error of the mean based on four biological replicates each calculated as the average of three technical replicates. For all genes, the expressions are normalized against the expression of actin. *Indicates that the expression at 0 h was statistically significant to the following time-points within the same group except 3 h (one-way ANOVA, *P* < 0.05). **Indicates that the expressions at 0 h were statistically significant to all of the following time-points within the same group (one-way ANOVA, *P* < 0.05).

Gene expressions were quantified during different stages of *A. niger* development. Preliminary results showed that due to the extractability of different structures, two RNA extraction protocols (see Methods) were required: The first included high force to break the tough cell walls of conidia and early germination structures; and, the second was more efficient for fragile structures. Notably, the second protocol was not vigorous enough to extract any RNA from spores (data not shown). Therefore, the “Plate” value in Figure 3 only represents gene expression in mycelia and conidiation structures, but not mRNA present in the produced conidia. cDNA was made with the mRNA as a template, and the relative expressions of the six putative trehalose synthesis genes, *tpsA*, *tpsB*, *tpsC*, *tppA*, *tppB* and *tppC*, were analyzed with real-time PCR.

The general expression pattern of the genes (Figure 3) was as follows: The expression was highest in still dormant conidia and had decreased by approximately 2-fold after 3 h incubation; after 6 h incubation there was a slight, but not significant, decrease; and, in 12 and 72 h mycelium the expression was very low. For *tpsB, tppA* and *tppC*, the expression was then up-regulated in sporulating colonies (5 days old), while it remained low for *tpsC* and *tppB*. One gene, *tppA*, deviated slightly from the described pattern: The decrease in expression after 3 h was not as profound as in the other genes, and a slight, but not significant, up-regulation could be seen in 72 h mycelium.

### Targeted gene deletions of six *Aspergillus niger* genes

To characterize the function of the six *A. niger* proteins, *tpsA, tpsB, tpsC, tppA, tppB* and *tppC* were all subjected to targeted gene deletions by replacing the gene with the *A. oryzae pyrG* resistance cassette. A double mutant, lacking the two adjacent genes *tpsB* and *tpsC* was also constructed. All deletion mutants were confirmed with PCR using both internal and flanking primers (data not shown).

**Figure 4:**
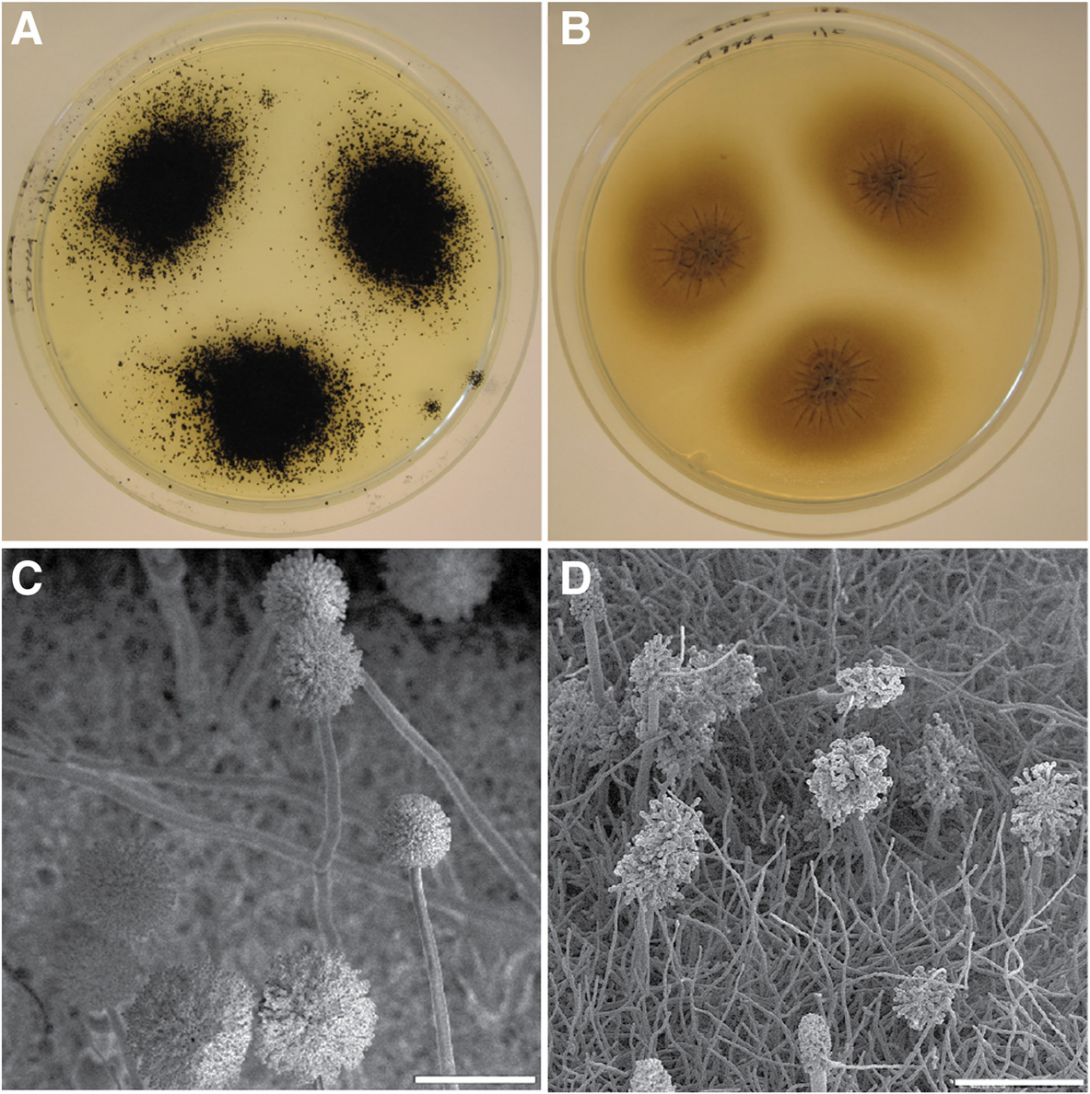
**Morphologies of cultures grown for 1 week on AMM.** Wild-type, left **(A and C)**, and *ΔtppA* right **(B and D)**. Size bars of SEM photos are 100 μm.

**Figure 5:**
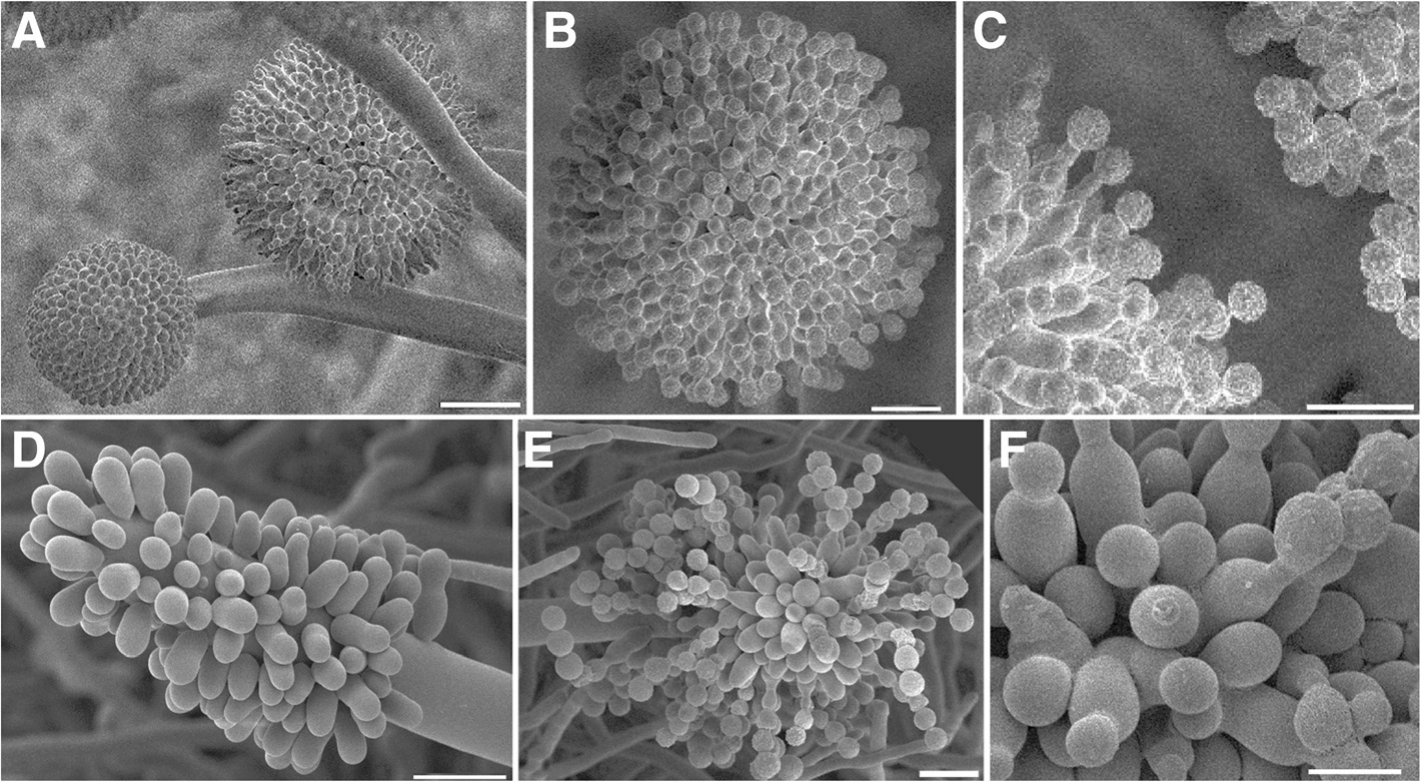
**Detailed morphologies of cultures grown for 1 week on AMM.** Wild-type, top **(A, B and C)**, and *ΔtppA* bottom **(D, E and F)**. Size bars: **A** = 20 μm, **B** = 10 μm, **C** = 10 μm, **D** = 10 μm, **E** = 10 μm, **F** = 5 μm.

With the exception of *ΔtppA*, all deletion mutants showed phenotypes similar to wild-type. When culturing the wild-types and mutants at temperatures ranging from 15°C to 37°C, no strain-dependent differences in growth rates or morphologies could be observed; at 10°C no growth was observed for any strain (data not shown). The *tppA* mutant showed a marked reduction in the number of conidia produced compared to the other strains, giving the colonies growing on plate a whitish, and with age, light brownish appearance, compared to the black wild-type (Figure 4A,B). This phenotype was retained during aging, and under all growth conditions. When comparing the spore densities, the wild-type, N402, yielded an average of 2.44 × 10^6^ (±0.045 × 10^6^) spores/mm^2^, whereas *ΔtppA* yielded an average of 4.40 × 10^3^ (±0.69 × 10^3^) spores/mm^2^, i.e. a 6 × 10^2^-fold reduction. Microscopic studies revealed that the conidiophores of *ΔtppA* had a clearly different appearance as is shown in Figure 4C and D. Most notably, vesicle swelling was almost completely absent and metulae were irregularly positioned (Figure 4C,D and Figure 5). However, the conidia produced showed similar size and ornamentation to wild-type (Figure 5C,F). In contrast to what has been reported in the corresponding mutant of *A. fumigatus*[22], it was not possible to restore wild-type morphology by growing *ΔtppA* on media containing an osmotic stabilizer, i.e. the described phenotype persisted in all growth conditions.

### Quantification of trehalose-6-phosphate and trehalose in wild-type and mutants

**Figure 6:**
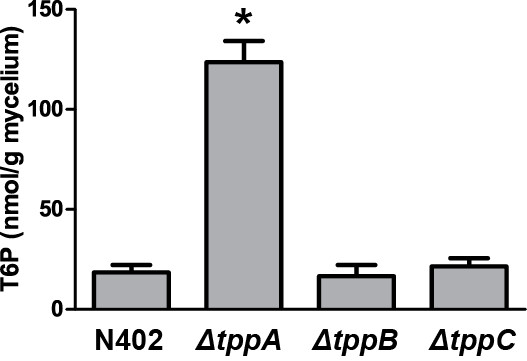
**Content of T6P in mycelium dry weight of wild-type and Tpp deletion mutants.** Error bars show standard error of the mean. In *ΔtppA*, the level of T6P was significantly higher compared to all other strains (one-way ANOVA, *P* < 0.05)

All three Tpp genes putatively encode the enzyme trehalose-6-phosphate-phosphatase. To investigate if this enzyme was absent in the Tpp deletion strains, the amount of trehalose-6-phosphate (T6P) in mycelia from wild-type, *ΔtppA*, *ΔtppB* and *ΔtppC* were analyzed. There were no significant differences in T6P levels between wild-type, *ΔtppB* or *ΔtppC*. In *ΔtppA*, however, T6P was clearly accumulated; the mycelium from this strain contained an average of 124 nmol T6P per gram dry weight compared to 18 nmol in the wild-type (Figure 6).

**Figure 7:**
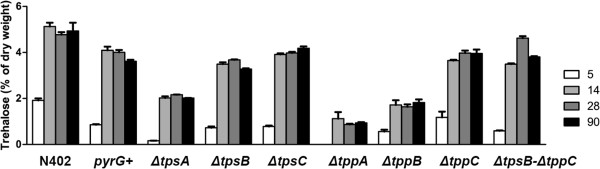
**Trehalose content in mutant and wild-type conidia of different age.** The numbers to the right represent how many days the colony had grown on AMM plates before conidia were harvested and analysed. Error bars show standard error of the mean.

To elucidate how specific gene products influence the trehalose content of *A. niger* conidia in different stages of maturation, conidia were harvested from control and mutant strains after 5, 14, 28 and 90 days. In these and the following stress experiments, in addition to the wild-type N402 strain, we also included a *kusA* deficient strain with a repaired *pyrG* gene, *pyrG +* [28] as a control with identical genetic background as the *tps* and *tpp* deletion mutants. The dormant conidia were extracted and the trehalose levels analyzed and expressed as percentage of conidial dry weight (Figure 7). For *ΔtppA* it was not possible to analyze the trehalose content of 5 day conidia, as insufficient conidia were produced. For the other strains, a significant increase in trehalose was detected between the two first time points tested, 5 and 14 days. During further aging of conidia, between 14 and 90 days, no significant changes in trehalose levels were detected (with the exception of *ΔtpsB-ΔtppC* where the level in 28 day old conidia was 4.6% compared to 3.5 and 3.8 in 14 and 90 day old conidia, respectively).

At all time points, conidia from all mutant strains contained significantly less trehalose compared to wild-type conidia (again, with the exception of *ΔtpsB-ΔtppC* 28 days). When comparing the deletion mutants to the other control strain, *pyrG*+, significantly lower levels of trehalose were detected in strains *ΔtpsA*, *ΔtppA* and *ΔtppB*. After 14 days of maturation the conidial trehalose level was 50% lower in *ΔtpsA* compared to *pyrG+*, and 73 and 60% lower in *ΔtppA* and *ΔtppB*, respectively. For *ΔtpsA* and *ΔtppA*, the reduction was significant at all time points tested, and for *ΔtppB*, the difference was significant in 14, 28 and 90 day old conidia but not after 5 days.

Among the deletion mutants with wild-type like phenotypes, i.e. when excluding *ΔtppA*, *ΔtppB* had the lowest overall trehalose content. After 14 days of incubation, the trehalose level was 1.7% of conidial dry weight compared to 5.1 and 4.1% in wild-type N402 and *pyrG*+, respectively. Although the conidial trehalose content was consistently lower in *ΔtppA*, the extremely low number of spores produced made this strain unsuitable for studies on conidial survival. Therefore, *ΔtppB* was, due to its wild-type morphology, selected for additional studies to reveal whether or not a normal internal trehalose level has any impact on stress survival and growth.

### Confirmation and further characterization of ***ΔtppB***

**Figure 8:**
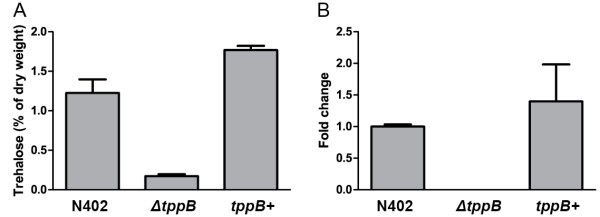
**Trehalose content of mycelium (A) and relative expression of *tppB *(B).** Error bars show standard error of the mean, based on three biological replicates, and for qPCR each biological replicate was calculated as the average of three technical replicates.

Before subjecting the *tppB* deletion mutant to stress, a few confirmatory experiments were performed to ensure that the lowered trehalose content was a consequence of the deleted gene: A new deletion mutant of *tppB*, *ΔtppB2*, was generated using MA169.4 as parent strain, and on a selected transformant the *ΔkusA* gene was restored using acetamide. Analysis of trehalose content in 14 day old conidia from this new mutant showed that they were as low as in *ΔtppB* (1.54 ± 0.1% of conidia dry weight in *ΔtppB2* versus 1.72 ± 0.5% in *ΔtppB*). Moreover, the deletions mutants were complemented by transformation of an autonomously replicating plasmid carrying the gene for hygromycin resistance as well as an intact copy of the *tppB* gene. Putative transformants were selected on hygromycin plates. The presence of the construct was confirmed using PCR and plasmid rescue (data not shown). In a previous study we discovered that, when using this methodology, only a fraction of conidia carry the plasmid [28]. This was also valid for *tppB +* conidia, where only a few percent germinated on hygromycin media (data not shown). Therefore, the mycelial trehalose content was measured after growth in liquid AMM media supplemented with hygromycin – under a continuous selection pressure to retain the plasmid, we showed that in *tppB +* the amount of trehalose was restored to wild-type level (Figure 8A). The expression of *tppB* was examined in mycelium from wild-type, *ΔtppB* and *tppB*+. In the deletion mutant, no expression was detected, whereas in the complemented strain, the levels were in the same range as in the wild-type (Figure 8B). From these experiments we concluded that the deletion of *tppB* causes the lowered trehalose levels in *ΔtppB*. However, since the plasmid carrying the wild-type version of the gene was lost in most conidia, the *tppB*+ strain was not included in the following experiments.

**Figure 9:**
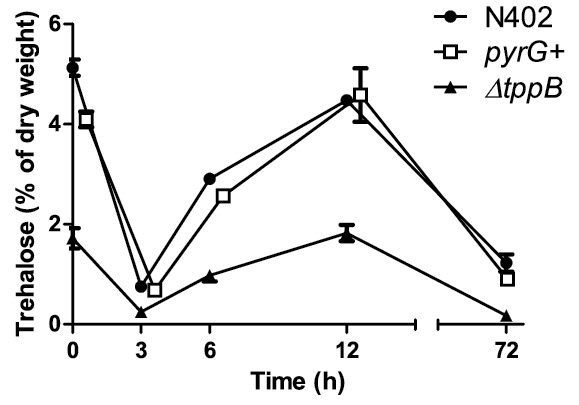
**Concentration of trehalose during outgrowth of wild-type, *pyrG +*** **and *ΔtppB *conidia.** Note the scale break between 12 and 72 h and that *pyrG +* observations are horizontally offset to avoid visual overlap. The error bars represent the standard error of the mean. The level of trehalose in *ΔtppB* was significantly different compared to wild-type for all time points except 3 h (two-way ANOVA, *P* < 0.0001 at 0, 6 and 12 h, and *P* < 0.01 at 72 h).

**Figure 10:**
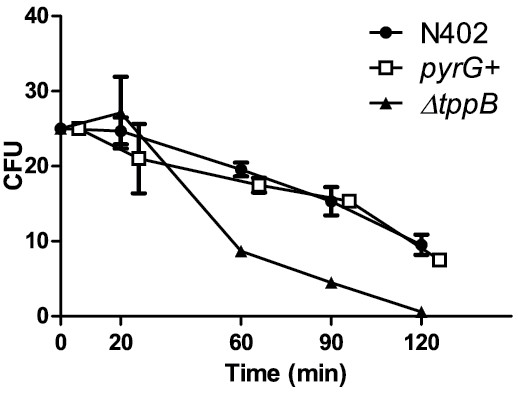
**Viabilities of dormant *A. niger *conidia after subjection to heat stress.** Conidia were held at 55°C for 20, 60, 90 and 120 min. For all strains, the numbers of counted colonies were normalized to 25 at time = 0 min to avoid differences in numbers of assayed spores. Note that *pyrG +* observations are horizontally offset to avoid visual overlap. There were no significant differences between the control strains (N402 and *pyrG+*). Except for the two first time points, the viability of *ΔtppB* was significantly lower than in N402 (two-way ANOVA, *P* < 0.01 in 60 and 90 min, and *P* < 0.05 in 120 min).

To evaluate the importance of trehalose as a stress protectant, the trehalose contents of the *ΔtppB* mutant and the control strains were analyzed in early stages of germination, and were subjected to lethal and sub-lethal heat and oxidative stress as well as sub-lethal salt and acid stress. The trehalose levels in *ΔtppB* followed the same pattern of breakdown and re-synthesis as in the control strains, but they were consistently lower in accordance with the lower initial value (Figure 9). Dormant conidia of *ΔtppB* were significantly less tolerant to heat stress compared to the control strains; After 60 min of heat stress, the survival of *ΔtppB* was 35% compared to 78% in wild-type. After an additional 60 min, the survival of *ΔtppB* further decreased to 2% compared to 38% in wild-type. (Figure 10). These experiments were repeated with the new independent deletion mutant, *ΔtppB*2, and the results were identical to those for *ΔtppB* (data not shown). For the other stressors tested, benzoic acid, NaCl and H_2_O_2_, as well as long-term viability where conidia were stored in water at 4°C for a total of 8 weeks, no significant differences between the mutant and the control strains could be detected (data not shown).

## Discussion

In this project we have studied six genes with a putative role in trehalose synthesis in *A. niger*: *tpsA*, *tpsB*, *tpsC*, *tppA*, *tppB* and *tppC*. All six genes encode homologous proteins and no similar gene products within the *A. niger* genome could be detected. Three proteins, TpsA, TpsB and TpsC, have previously been identified as orthologs to the yeast protein Tps1. As the orthologs are conserved in related species, it is plausible that there is a functional differentiation between the paralogs, e.g. one paralog could be essential for trehalose synthesis in conidia, whereas another paralog is strictly induced by stress. This assumption is in line with the previous observation in *A. niger* where the expression of *tpsB* is stress-induced whereas *tpsA* is constitutively expressed [23], although our data also suggest that *tpsB* has a role during differentiation (see Figure 3). When deleting the trehalose-phosphate-synthase paralogs, only *ΔtpsA* displayed a reduced trehalose content. The lower level in this mutant is in line with a previous report using a different target strain and deletion procedure [23]. In the related fungus, *A. fumigatus*, a *tpsA*/*tpsB* double deletion resulted in a strain with depleted trehalose content, and in the same study, it was shown that the expressions of *tpsC* and *–D* were very low at all time points [12]. These authors evaluated their expression data using RNA extracted from hyphae, and in the present study, the *A. niger tpsC* was expressed at very low levels at 72 h. Thus the results from the two fungi are not contradictory, and most likely an *A. niger tpsA/tpsB* deletion mutant would also have a depleted trehalose content. The results from *A. niger* and *A. fumigatus* are also in accordance with findings in *A. nidulans* where deletion of *tpsA* resulted in depleted trehalose content [11], as that species does not have the *tpsB* paralogue. A conclusion from studying the trehalose content from these three species is that TpsA is the most important trehalose-phosphate-synthase under normal conditions, but lack of the *tpsA* gene can be fully compensated by TpsB in *A. fumigatus* and partly by at least one of TpsB or TpsC in *A. niger*, but not by TpsD in *A. nidulans*.

The deletion mutant with the most distinctive characteristics in our experiments was *ΔtppA*, i.e. with an abnormal morphology and reduced levels of both trehalose-6-phosphate and trehalose. The altered morphology of the strain is probably due to toxicity of T6P as indicated for the corresponding deletion mutant in *A. fumigatus*[22]. However, in *A. niger* as well as *A. fumigatus* and *A. nidulans*[12,25], mutants of *tppA* are not totally lacking in trehalose. Therefore, it is possible that under specific conditions, e.g. when TppA is absent, TppB, and also TppC where present, may contribute to some T6P activity. Another possibility is that the sugar can be synthesized by proteins other than Tps/Tpp, e.g. the Trehalose Phosphorylase pathway, for which putative genes have been identified and partially characterized in *N. crassa*[40] and *A. fumigatus*[22] and also exist in *A. niger* (ANI_1_2720024). However, it is possible to generate mutants, within the homologous Tps/Tpp group, in *A. fumigatus* and *A. nidulans* that totally lack trehalose [11,12]. Therefore, we believe that this is the only active trehalose synthesis pathway in Aspergilli. However, internal trehalose contents may not solely be dependent on the presence and expression of these six genes, as in *S. cerevisiae* there is a strong linkage between trehalose synthesis and the degrading trehalases [41] as well as evidences of posttranscriptional activation of the genes involved in trehalose metabolism [42,43].

Besides a putative phosphatase activity, TppB and TppC may have similar biological roles as the yeast proteins Tps3 and Tsl1, which also contain phosphatase domains – in yeasts, deletion of both genes is necessary before some reduction in internal trehalose content can be observed [17]. It is intriguing that *tpsB* and *tppC* are linked on the chromosome. We cannot explain why the conidial trehalose content in this double mutant was significantly higher after 28 days, but based on the expression patterns (see Figure 3), it is possible that the expression of the two genes are regulated by the same factors. In addition to the above-mentioned observations, some conclusions can be drawn from the gene expression data: All identified genes were expressed, indicating that the paralogs are not inactive duplicates. For *tpsC* and *tppB*, the expressions were consistently low after 6 h, indicating that the two genes may be regulated by the same mechanism. This assumption is supported by a previous observation using *A. oryzae* arrays where the *tpsC* and *tppB* orthologs were down-regulated in a deletion strain of *atfA*, a gene encoding a transcription factor [44]. To our knowledge, two previous studies describing the expression of trehalose synthesis genes in *A. niger* during germination, using microarray technology, or in combination with RNA sequencing, have been published [29,45]. With the exception that van Leeuwen and co-workers [29] saw a drastic drop after 2 h and then a gradual up-regulation of *tpsA* and *tpsB,* those results are in line with our findings.

The extensive measurements of internal trehalose indicate that the trehalose contents, for all strains, were low in 5 day old conidia, significantly elevated in 14 day old conidia, and then maintained at the value of 14 days (Figure 7). A plausible hypothesis is that conidia of *A. niger* reach full maturity, at least in terms of trehalose accumulation, sometime between 5 days and 2 weeks. Consequently it is not advisable to perform stress experiments on young conidia because their trehalose content is not necessarily typical for the final level, especially not in *kusA* deficient strains that seem to have slower conidial maturation in terms of trehalose content.

We found that 2 week old conidia of *ΔtppB* were more susceptible to heat shock than wild-type conidia, indicating that trehalose protects the spores from thermal stress. These results are in line with earlier studies in *Aspergillus* species [11,12,23]. However, in contrast to results from *A. fumigatus* and *A. nidulans*, we could not detect any increased sensitivity of *ΔtppB* to oxidative stress [11,12], salt or acid stress, or any decreased viability after long term storage. It should be noted that unlike *ΔtppB* in our experiments, which harbored approximately one third of wild-type trehalose content, the *A. fumigatus* and *A. nidulans* mutants were totally depleted of trehalose.

In *S. cerevisiae* it has been shown that, using a two-hybrid assay, the four homologous proteins physically interact. When repeating the experiments using the six identified *A. niger* proteins, we could observe interactions for four of six proteins. These results suggest that TppA and TpsA-C form a complex, while the phylogenetically more distant proteins, TppB and TppC, are present outside the complex. However, due to the experimental limits, it is possible that neither TppB nor TppC was correctly folded and therefore not interacting. It is notable that in *S. cerevisiae*, a truncated version of Tsl1 was necessary for the success of the interaction experiments [40], in contrast to our experiment in which we only used full-length proteins.

## Conclusions

To conclude, in this study novel information about the six gene products involved in trehalose synthesis in *A. niger* has been generated. When characterizing deletion mutants, lack of the most conserved trehalose phosphate synthase *tpsA*, the trehalose phosphate phosphatase *tppA,* or the previously non-characterized *tppB,* resulted in lower trehalose contents. An additional insight is that the components in a putative trehalose synthesis complex differ among the Aspergilli, but some gene products are common throughout the fungal kingdom.

## Competing interests

The authors declare the absence of competing interests.

## Authors’ contributions

ÅS performed the majority of the laboratorial work. ÅS and PM performed all experiments with exception of the RNA extraction from dormant conidia and conidia in early stages of germination, performed by MRL, and the SEM studies, performed by JD. ÅS and PM conceived and designed the study and wrote the manuscript. All authors read and approved the final manuscript.
